# Overexpression of miR-483-5p/3p cooperate to inhibit mouse liver fibrosis by suppressing the TGF-β stimulated HSCs in transgenic mice

**DOI:** 10.1111/jcmm.12293

**Published:** 2014-05-06

**Authors:** Fuyuan Li, Ning Ma, Ruiqi Zhao, Guodong Wu, Yanfen Zhang, Yu Qiao, Dong Han, Ya Xu, Ying Xiang, Bingzhu Yan, Jianfeng Jin, Guixiang Lv, Lei Wang, Changqing Xu, Xu Gao, Shanshun Luo

**Affiliations:** a Department of Biochemistry and Molecular Biology, Harbin Medical UniversityHarbin, China; b Translational Medicine Center of Northern ChinaHarbin, China; c Department of Laboratory Diagnosis, Affiliated Hospital of Harbin Medical UniversityHarbin, China; d Department of Cell Biology, Medical School, Yangtze UniversityJingzhou, China; e Department of Infectious Diseases, Second Affiliated Hospital of Harbin Medical UniversityHarbin, China; f Department of Pathophysiology, Harbin Medical UniversityHarbin, China; g Basic Medical Institute of Heilongjiang Medical Science AcademyHarbin, China; h Key Laboratory of Cardiovascular Medicine Research (Harbin Medical University), Ministry of EducationHarbin, China; i Department of Gerontology, First Affiliated Hospital of Harbin Medical UniversityHarbin, China

**Keywords:** liver fibrosis, microRNA, HSCs, transgenic mice

## Abstract

The transition from liver fibrosis to hepatocellular carcinoma (HCC) has been suggested to be a continuous and developmental pathological process. MicroRNAs (miRNAs) are recently discovered molecules that regulate the expression of genes involved in liver disease. Many reports demonstrate that miR-483-5p and miR-483-3p, which originate from miR-483, are up-regulated in HCC, and their oncogenic targets have been identified. However, recent studies have suggested that miR-483-5p/3p is partially down-regulated in HCC samples and is down-regulated in rat liver fibrosis. Therefore, the aberrant expression and function of miR-483 in liver fibrosis remains elusive. In this study, we demonstrate that overexpression of miR-483 *in vivo* inhibits mouse liver fibrosis induced by CCl_4_. We demonstrate that miR-483-5p/3p acts together to target two pro-fibrosis factors, platelet-derived growth factor-β and tissue inhibitor of metalloproteinase 2, which suppress the activation of hepatic stellate cells (HSC) LX-2. Our work identifies the pathway that regulates liver fibrosis by inhibiting the activation of HSCs.

## Introduction

The liver is one of the most vital organs in humans because of its role in metabolism, nutrition and biotransformation. Additionally, the liver is the most susceptible organ to damaging factors, such as alcohol, viruses and toxins, which induce liver diseases, such as hepatitis, liver fibrosis, cirrhosis and hepatocellular carcinoma (HCC) [1]. Hepatocellular carcinoma is the fifth most prevalent cancer in the world and has a high mortality rate. However, liver fibrosis is a reversible wound-healing response to either acute or chronic cellular injury that reflects a balance between liver repair and scar formation [2]. Therefore, research focusing on the molecular mechanisms of liver disease, particularly liver fibrosis, may identify novel targets for gene therapy.

Although a number of cell signalling, viral and growth factors have been associated with the development of liver disease, the precise regulation of gene expression remains unknown. A microRNA (miRNA) is a small (18–24 nucleotides) non-coding RNA that regulates gene expression by binding to its target mRNA to interfere with translation [3]. miRNAs target and regulate essentially all biological processes and cell types, including those within the liver. Numerous reports have demonstrated that alterations in the expression of intracellular and extracellular miRNAs correlate with various liver diseases, including viral-related hepatitis, non-alcoholic steatohepatitis, liver fibrosis and HCC [4,5]. miR-122 has been the most thoroughly studied miRNA with regard to liver pathophysiology. miR-122 is highly abundant in the human liver and is essential for HCV replication [6,7]. miR-29 regulates the activation of hepatic stellate cells (HSCs) mediated by transforming growth factor-β (TGF-β) [8]. In addition, miRNAs in the serum or blood can be used as potential diagnostic biomarkers of liver disease [9,10]. However, the role of an individual miRNA in the progression of liver disease and in cell–cell interactions in the liver remains unknown.

miR-483-5p and miR-483-3p were identified from a human embryonic liver and are generated from the same pre-cursor miRNA, which is derived from the second intron of the insulin-like growth factor 2 gene (Igf2) [11]. Reports have suggested that some intragenic miRNAs co-express and cooperate with their host genes, but that other miRNAs do not [12]. Igf2 overexpression was observed during the progression from liver fibrosis to HCC, and IGF2 promoted proliferation and carcinogenesis [13]. In addition, miR-483 is up-regulated in approximately half of human tumours [14], including adrenocortical carcinoma and HCC [15,16], and its oncogenic targets, PUMA, CTNNB1, IGF1R, have been identified. Our previous study showed that miR-483-5p promoted the proliferation of a mouse HCC cell line [17]. These data suggest that miR-483 is a partner of its host gene Igf2. However, recently, miR-483 was reported to be down-regulated in activated rat primary HSCs induced by choline-deficient ethionine supplementation [18]. A study by Veronese *et al*. showed that miR-483-3p is down-regulated in HCC by its own transcriptional region [19], and Wang *et al*. reported that miR-483 is down-regulated in HCC [20]. The variable expression of miR-483 causes the development of HCC from liver fibrosis, which has been previously suggested to be a continuous process.

The objective of our study was to thoroughly and systematically investigate the function of miR-483 *in vivo*. Therefore, we engineered pre-miR-483 overexpressing transgenic mice under the control of the cytomegalovirus early enhancer/chicken β-actin (CAG) promoter. We found that overexpression of pre-miR-483 inhibits CCl_4_-induced liver fibrosis by targeting platelet-derived growth factor-β (PDGF-β) and tissue inhibitor of metalloproteinase 2 (TIMP2) in the activation of HSCs. Finally, we found overexpression of miR-483 induced mice liver carcinogenesis. Our findings address the function of miR-483 in liver fibrosis.

## Materials and methods

### Transgenic mice

To generate the miR-483 transgenic mice, pre-miR-483 with a flank sequence fragment was PCR-amplified from mouse genomic DNA with cloning primers (Data S1). Pre-miR-483 was cloned into the EcoR I site of a pCAGGs expression vector, which contained the chicken β-actin promoter. TG mice were generated by pronuclear injection of the transgene into the C57BL/6 strain. Genomic DNA isolated from the tail was analysed by PCR by using specific primers (Data S1).

### Cell co-culture

For the direct co-culture assay, two cell lines, HL7702 and LX-2, were treated with TGF-β, mixed and seeded at concentrations of 1.5 × 10^6^ cells per well for HL7702 cells and 1.0 × 10^6^ cells per well for LX-2 cells in 3.5-cm plates.

For the indirect co-culture assay, polycarbonate membrane inserts in multidishes (Nunc, Beijing, China) were used. The LX-2 cells (0.5 × 10^5^ cells per well) were seeded in the lower 3.5-cm plates, and the HL7702 cells (the 1.0 × 10^5^ cells per well) with transfected miR-483 were seeded in the upper transwell inserts. The cells were pre-cultured for 16 hrs and then treated with TGF-β for the indicated times.

The secretion of miR-483 was observed after the culture media was changed 48 hrs after the transfection of fluorescently labelled miR-483 after the cells were washed three times with PBS. The presence of miR-483 was determined 48 hrs after the co-culture of HCs and HSCs after the cells were washed three times with PBS (2 ml/wash). The transfection of the fluorescently labelled miR-483 was performed in the dark.

Other reagents and methods are described in the Data S1.

## Results

### miR-483-5p and miR-483-3p expression is reduced in CCL_4_-induced mouse liver fibrosis

**Figure 1:**
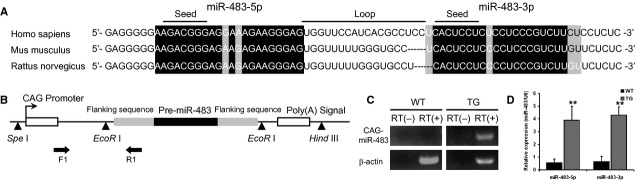
Production and characterization of miR-483. (A) Alignment of pre-miR-483 sequences from different species (primates and rodents). White characters indicate the pre-miR-483 sequence, black background indicates homology, and grey background indicates different nucleotides. (B) The construct for the miR-483 transgene. miR-483 was cloned between the two EcoR I sites, which puts the transgene under the control of the CAG promoter. The construct was then injected into the male pronuclei of the oocytes of pregnant C57BL/6 mice. (C) Reverse transcriptional PCR was used to detect the expression of the transgene in the liver. (D) The expression of the transgene was assessed by qRT-PCR on total RNA extracted from the livers of 2-month-old mice. **P < 0.01.

miR-483-5p and miR-483-3p originate from the pre-miR-483 locus. These miRNAs have high homology in humans, mice and rats (Fig.1A). miR-483 was reported to be down-regulated in activated rat primary HSCs. To determine the level of miR-483 in liver fibrosis *in vivo*, we used the well-established carbon tetrachloride (CCl_4_) treatment model to induce hepatic fibrosis in mice (Fig. S1A). As expected, 8 weeks of CCl_4_ administration caused hepatic fibrosis in the livers of the treated mice as assessed by serum detection, Masson staining and the expression of α-SMA (smooth muscle actin) at the translational level (Fig. S1B–D). miR-483-5p and miR-483-3p were compared in the fibrotic livers from mice with either CCl_4_ treatment or olive oil alone (control). Both miR-483-5p and miR-483-3p showed significantly lower expression compared to the control animals. The expression changes of the two miRNAs positively correlate with the degree of liver fibrosis (Fig. S1E). However, miR-483-5p, but not miR-483-3p, is down-regulated only in thioacetamide (TAA)-induced liver fibrosis, which may result from its own transcriptional-regulation region [19] or the difference between TAA and CCl_4_. Therefore, we determined that the level of miR-483 is down-regulated during CCl_4_-induced liver fibrosis in mice.

### Overexpression of pre-miR-483 *in vivo* inhibited CCl_4_-induced liver fibrosis

**Figure 2:**
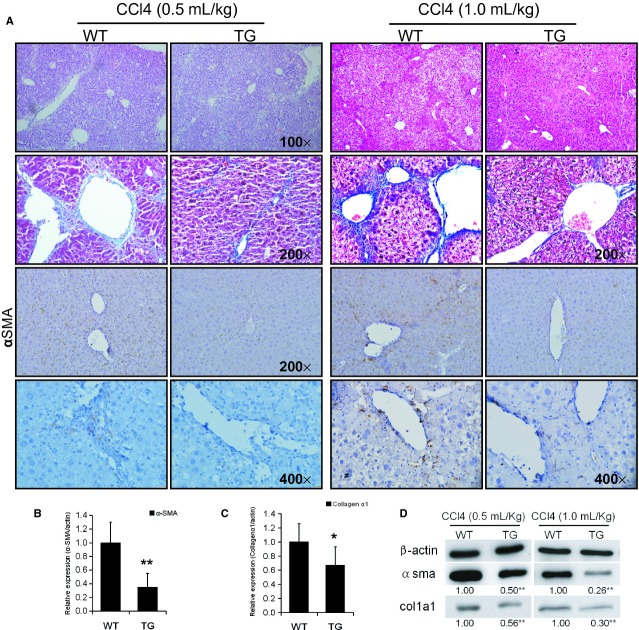
Overexpression of pre-miR-483 inhibits CCl_4_-induced liver fibrosis in transgenic mice. (A) Haematoxylin and eosin and Masson staining of liver sections from transgenic and wild-type mice (×100/×200), immunohistochemical analysis of α-SMA and collagen1α1 (×200/×400). The results show increased collagen deposition in the transgenic mice compared to the wild-type mice, and the degree of deposition correlates with the dose of CCl_4_. The level of α-SMA and collagen1α1 of the WT mice are higher than in the transgenic mice. (B) The transcriptional level of α-SMA in liver. The transgenic mice presented with less α-SMA in the liver fibrosis induced by CCl_4_ (0.5 ml/kg). (C) The mRNA expression of collagen1α1 as determined by qRT-PCR. (D) The translationl level of α-SMA and collagen1α1 in WT and transgenic mice liver treated with low and high dose of CCl_4_. Overexpression of miR-483 reduced the up-regulation of α-SMA and collagen1α1 in mouse liver induced by CCl_4_. *P < 0.05, **P < 0.01.

To evaluate the role of miR-483 *in vivo*, we engineered pre-miR-483 transgenic mice, the expression of which is driven by the CAG promoter (Fig.1B). The transgenic mouse liver overexpressed miR-483-5p and miR-483-3p (Fig.1C and D). In the transgenic mice, the administration of CCl_4_ for 8 weeks caused less collagen deposition and an even greater reduction in α-SMA, a marker of HSC activity, compared to the CCl_4_-induced WT mice at both the transcriptional and translational level (Fig.2A). These changes were more prominent in the group treated with high dose CCl_4_ (Fig.2A). Collectively, these data demonstrate that overexpression of pre-miR-483 may inhibit liver fibrosis and that miR-483 is a protective factor against liver fibrosis.

### miR-483-5p and miR-483-3p inhibit TGF-β stimulated HSC LX-2 cells

**Figure 3:**
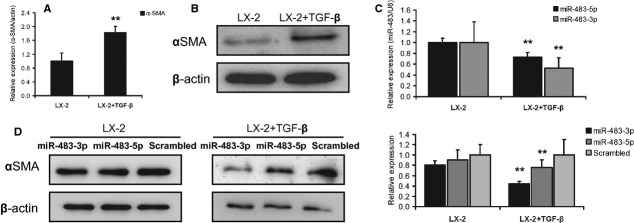
miR-483-5p and miR-483-3p inhibit transforming growth factor-β (TGF-β) stimulated LX-2 cells. (A) Expression of α-SMA at the transcriptional level in quiescent *versus* TGF-β stimulated LX-2 cells. (B) Expression of α-SMA at the translational level in quiescent *versus* TGF-β stimulated LX-2 cells. (C) qRT-PCR analysis for miR-483-5p and miR-483-3p was performed with RNA extracts from quiescent and activated hepatic stellate cells (*n* = 4). (D) Regulation of α-SMA proteins after miR-483 transfection. (E) Immunofluorescence for α-SMA proteins after miR-483 transfection. Three independent experiments were performed; **P < 0.01.

The activation of HSCs is a key process during liver fibrosis *in vivo*. Because we observed the inhibition of α-SMA in miR-483 transgenic mice, we investigated the role of miR-483 in the activation of HSCs. First, stimulation of the human HSC cell line LX-2 with recombinant TGF-β, a major cytokine involved in HSC activation (Fig.3A and B), led to an increase in TIMP2 and PDGF-β (Fig. S2A) and significant decrease in miR-483 expression (Fig.3C). These results are consistent with the results observed for CCl_4_-induced liver fibrosis in transgenic mice. We then transfected either miR-483 or a control sequence into the TGF-β stimulated LX-2 cells. α-SMA was down-regulated by overexpression of miR-483 at the translational level (Fig.3D). These results are consistent with the role of miR-483 *in vivo*. Our data suggest that overexpression of miR-483 regulates liver fibrosis by inhibiting the activation of HSCs in transgenic mice. Next, we identified the targets of miR-483 in this regulatory process.

### miR-483-5p and miR-483-3p cooperatively target PDGF-β and TIMP2

**Figure 4:**
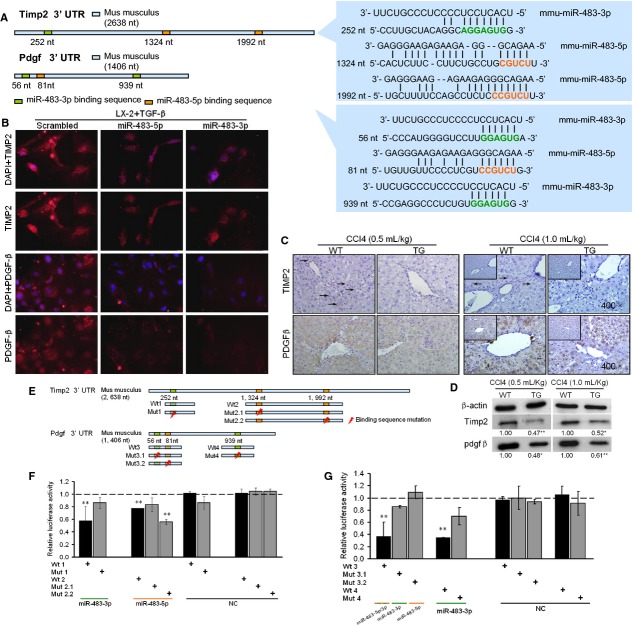
miR-483 down-regulates platelet-derived growth factor-β (PDGF-β) and tissue inhibitor of metalloproteinase 2 (TIMP2). (A) The sequence sites of miR-483 and the binding UTR of PDGF-β and TIMP2. (B) miR-483 inhibits the expression of PDGF-β after transfection *in vitro*. Immunohistochemistry (C) and Western blotting (D) for TIMP2 and PDGF-β in transgenic and wild-type mice liver. Overexpression of miR-483 decreased the translation of TIMP2 and PDGF-β. (E) The schematic diagram shows luciferase constructs containing each putative miR-483 binding site with or without mutation. (F and G) The luciferase activity assay with the mouse TIMP2 and PDGF-β UTRs. miR-483-3p and miR-483-5p reduced the expression of TIMP2 and PDGF-β by targeting their UTR; *P < 0.05, **P < 0.01.

To further elucidate the molecular mechanism by which miR-483 regulates the activation of HSCs, we used bioinformatics software to predict the targets of miR-483. The predicted targets of miR-483 include pro-fibrosis and anti-fibrosis genes in human and mice (Table S1). Of the targets, PDGF-β and TIMP2 are key anti-fibrosis molecules and are overexpressed in during HSC activation. Interestingly, the putative miR-483 binding sites in the 3′ UTR of the PDGF-β mRNA in both humans and mice share multi-binding sites in each UTR, but not in TIMP2 (Fig.4A, Fig. S3).

To verify the targets of miR-483 in the activation of HSCs, we first determined the expression of the potential targets PDGF-β and TIMP2 after transfection with the miR-483 mimics or control into LX-2 cells, human HSC cell line. PDGF-β and TIMP2 were down-regulated by miR-483 (Fig.4B; Western blotting, Fig. S2B), although TIMP2 cannot be predicted as th target of miR-483-3p. This suggested an indirect effect that miR-483-3p regulated the expression of TIMP2 in LX-2 cells. In addition, PDGF-β and TIMP2 protein expression was decreased in the livers of the CCl_4_-treated miR-483 transgenic mice compared to the wild-type littermates (Fig.4C and D), which is consistent with the conclusion that miR-483 acts as a negative regulator of PDGF-β and TIMP2 *in vitro*. To further test whether the putative miR-483 target sequences in the PDGF-β and TIMP2 3′ UTRs mediate the translational repression of PDGF-β and TIMP2 in mice, we inserted the mice 3′ UTR of either the PDGF-β or TIMP2 transcript, which contained either the binding sequences (56-76, 80-101, 939-959 for PDGF-β and 252-273, 1324-1347, 1992-2015 for TIMP2, See Fig. S4) or a mutant sequence, into a luciferase expression plasmid (Fig.4E). This was then transfected into HEK293T cells. Increasing amounts of miR-483 resulted in a decrease in luciferase activity, whereas the control sequence had no effect (Fig.4F and G). Interestingly, the binding sequence of the UTR (1324-1347) seemed more important than the UTR (1992-2015) for miR-483-5p in TIMP2 (Fig.4F). Altogether, our results show that overexpression of miR-483 inhibits liver fibrosis, in part by suppressing PDGF-β and TIMP2 during the activation of HSCs in transgenic mice.

### Overexpression of miR-483-5p in HCs reduces the expression of artificial target in HSCs

Sixty-seven per cent of the cells in the liver are hepatocytes [21]. In our miR-483 transgenic mice, ubiquitous expression of miR-483 resulted from the CAG promoter. To further elucidate the inhibitory effect of miR-483 on liver fibrosis in miR-483 transgenic mice, we determined whether HCs that overexpress miR-483 could regulate the activation of HSCs. First, we performed a transfection assay with carboxyfluorescent labelled miR-483 by using the hepatocyte cell line HL7702. We observed that the green fluorescence among HL7702 cells (Fig. S5A). Then, a direct co-culture assay was performed with the two cell types (Fig. S6A and B). The green fluorescence was observed in the HSCs (LX-2) activated by TGF-β after the cells were washed with PBS (Fig. S5B). The similar result was observed in the LX-2 which has not been activated (Fig. S7). We distinguished the HL7702 and LX-2 with regard to morphology (Fig. S8). Finally, we built a reporter plasmid containing a reporter gene GFP (green fluorescent protein) and a double copy anti-sense sequence of miR-483-5p (Sponges-miR-483-5p, S-483) downstream of GFP gene which could be served as an artificial target of miR-483-5p. A direct co-culture assay was performed; the results showed that overexpression of miR-483 in HL7702 cells could reduced the expression of GFP-S-483 in LX-2 (Fig. S9). However, we failed to observe the distinguished role of overexpression of miR-483 in HL7702 cells on the α-SMA protein level in LX-2 by a direct co-culture assay (Fig. S6C). Although our results suggested an intercellular transfer of miR-483 between HL7702 and LX-2 cells, we still lack sufficient evidence of the role of miR-483 in different cells. The role of overexpression of miR-483 in HCs on the activation of HSCs in the miR-483 transgenic mice needs further research.

### Dysregulation of miR-483 in mice liver carcinogenesis

Because fibrosis can progress to HCC, we detected spontaneous tumours in the miR-483 transgenic mice. This result suggests that overexpression of miR-483 for 12 months promotes HCC carcinogenesis (Fig. S10A). Overexpression of miR-483 may cause DEN-induced carcinogenesis (Fig. S10B and C). The suppressor of cytokine signalling 3 (Socs3) is a verified functional target of miR-483-5p and, was down-regulated in TG mouse livers (Fig. S10D and E). Although our finding demonstrated the down-regulation and function of miR-483 in mouse liver fibrosis, carcinogenesis was also induced. These results might come from the dysregulation in different cells types (Fig. S11).

## Discussion

It has been previously suggested that the regulation and function of miRNAs is highly correlated with liver disease [5]. Although miR-483 was identified in foetal liver [22] and overexpressed in liver cancer [14,15], it is down-regulated in activated rat primary HSCs [18]. In addition, this study showed that miR-483-3p has its own upstream transcriptional-regulation region [19], and miR-483-5p and miR-483-3p present with differential expression patterns in developing human brains [23]. Until recently, genetically modified animals for functional miR-483 research were not available. Therefore, we focused on the role of miR-483 in human development and disease, particularly liver disease. We first choose the CAG promoter for the transgenic mice because miR-483 has lbeen detected in the liver tissue in previous studies. The changes may be a result of the many types of liver cells.

Interestingly, miR-483 is embedded in the second intron of the Igf2 gene, which is overexpressed in liver fibrosis and HCC [24]. As an intragenic miRNA, miR-483 may affect the function of its host gene. Reports have shown that miR-208 and miR-140 affect their host genes 25,26; however, miR-26 suppresses its host gene to regulate neurogenesis [27]. In our earlier studies, miR-483 was identified as an oncogenic factor in cancer cells, which is similar to its host gene Igf2 [17]. However, miR-483 was not an oncogenic factor in human umbilical vein endothelial cells [28]. In our current study, miR-483 and the host gene play different roles. Moreover, miR-483 belongs to the Igf2-H19 locus, which is an important imprinting region in development and carcinogenesis [29]. The long non-coding RNA H19 functions as a precursor of miR-675, which in turn suppresses Igf1r [30,31]. miR-483 and miR-675 appear to be a pair of intragenic miRNAs that regulate the expression of this imprinting region. Further studies are necessary to address the regulation of this region.

Platelet-derived growth factor-β has been reported to be the most potent mitogen of HSCs, and the TIMP-2 gene has been reported to be dysexpressed and dysfunctional during the progression of chronic liver disease [32,33]. Therefore, the antagonism of PDGF-β and TIMP2 is a potential anti-fibrotic strategy. Interestingly, miR-483 is conserved in both humans and mice; however, the binding sites in the UTR of TIMP2 are not conserved (Fig. S3). Han *et al*. reported that the target of miR-483-3p, hMECP2, which contains a long UTR, is unique to humans [23]. Furthermore, Ghosh *et al*. and Berezikov reported that the UTRs of miRNAs are important in evolution [34,35]. Our study supports this conclusion. Our results show that PDGF-β may be an important target for future gene therapy in humans.

We explored the potential interaction between HCs and HSCs in miR-483 transgenic mice. Recently, miRNAs were identified that function in intercellular communication. For example, miR-150 is involved in heart disease *via* intercellular communication [36]. Microvesicles and exosomes were identified as the vector of delivering the miRNAs between the cells [37,38]. However, further studies are necessary to determine the mechanism by which miR-483 is delivered during liver fibrosis in the transgenic mice. Because of the loss of target specificity in current gene therapy regimens, research into the interactions between different cell types may lead to progress in identifying interfering molecules. Our results demonstrate that overexpression of miR-483 in HCs may inhibit liver fibrosis for future gene therapy.

Previous studies demonstrated that progression from liver fibrosis to HCC is a continuous process. Many cellular factors display continuous changes during this process. However, dysexpression of miR-483 occurred during this continuous process. Similarly, we observed that miR-199a is up-regulated in liver fibrosis [39] but down-regulated in HCC [40]. These observations suggested that different cell types should be evaluated individually in the development of liver fibrosis to HCC. These data suggest that a more complex molecular mechanism creates these continuous changes.

In this study, we provide evidence for an anti-fibrosis role of miR-483-5p and miR-483-3p during the progression of liver disease. However, the dysexpression of miR-483 in liver fibrosis and HCC might result from other mechanism, such as H19 gene and its intragenic miRNA miR-675.

In summary, our results reveal that miR-483-5p/3p overexpression inhibits the activation of HSCs in the liver both *in vitro* and *in vivo*. This effect might depend on at least two following pathways: (1) miR-483 inhibits the activation of HSCs by directly suppressing PDGF-β and TIMP2; and (2) HCs, which overexpress miR-483, might help to reduce the activation of HSCs in miR-483 transgenic mice. These miRNAs may be used clinically in the future to prevent with human liver fibrosis.

## Supporting Information

**Figure S1**. Down-regulation of miR-483-5p and miR-483-3p in CCl4- or TAA-induced liver fibrosis in mice. **Figure S2**. The expression of TIMP2 and PDGF at translational level after activition by TGF and overexpress miR-483. **Figure S3**. The UTRs of TIMP2 and PDGF-β of human and mice. **Figure S4**. Mutant UTR of TIMP2 and PDGF-β. **Figure S5**. (A) The carboxyfluorescent labelled miR-483 was observed in the cell culture media of HL7702 cells after the culture media was changed 48 hrs after the transfection and the cells were washed three times with PBS (Black arrowhead: carboxyfluorescent labelled miR-483). **Figure S6**. Direct and indirect cell co-culture of HL7702 and LX-2. **Figure S7**. The green fluorescence dot could be observed in LX-2 cells; the lower left: bright field, the lower right: fluorescence. **Figure S8**. The morphology of HL7702 and LX-2 cells. **Figure S9**. Co-culture assay of HL7702 transfected with miR-483-5p (Scrambled) and LX-2 transfected with RFP/GFP-S-483. **Figure S10**. Spontaneous tumours in miR-483 transgenic mice. **Figure S11**. Differential expression of miR-483-5p in several cell lines.

**Table S1**. Potential targets of miR-483 predicted by bioinformatics software.

**Data S1**. Primers and sequences.
