# Supplementary material for: Overexpression of miR-483-5p/3p cooperate to inhibit mouse liver fibrosis by suppressing the TGF-β stimulated HSCs in transgenic mice
Source: J Cell Mol Med. 2014 May 6;18(6):966–74. doi: 10.1111/jcmm.12293 (PMC4508137; doi:10.1111/jcmm.12293)
Supplement: Supplementary file 1 [file jcmm0018-0966-sd1.doc]

**Supplemental material------Methods and Materials**

**Mouse model of liver fibrosis**

All mice were housed, handled, and killed under the supervision of the Animal Care and Use Committee of Harbin Medical University. Eight-week-old mice (n = 5) were injected intraperitoneally with 100 mg/kg body weight of thioacetamide (TAA, Sigma) diluted in saline three times a week for six weeks. Control mice (n = 5) were injected with saline. As another liver fibrosis model, mice were given either a 0.5 mL/kg (low dose group) or 1.0 mL/kg (high dose group) dose of carbon tetrachloride (CCl_4_, sigma-aldrich, United States) or olive oil two times a week for eight weeks.

**Serological and histological analysis**

Serum biochemical studies, including AST and ALT analysis, were performed on the liver fibrosis mouse model. Resected liver tissue was processed as either paraffin sections. The paraffin sections (6 µm) were processed to allow H&E staining, Masson staining, and immunohistochemical studies. The immunohistochemical analysis used antibodies against α-SMA (Abcam, Beijing, China), collagen I (Cell Signaling Technology, Beijing, China), PDGF-β, TIMP2, and PDGF-βR (Santa Cruz Biotechnology, Beijing, China).

**Cell culture and transfection**

The human stellate cell lines LX-2 were maintained in D-MEM (Gibco, Beijing, China) with 10% foetal bovine serum, plated in 60-mm diameter dishes and cultured to 70% confluence. Huh-7, HL7702 (1640 culture media), and HepG2 cells were also maintained in D-MEM with 10% foetal bovine serum with 0.1% penicillin/streptomycin. LX-2 cells were then cultured in serum-free D-MEM with 0.2% BSA for 48 hours prior to TGF-β (Sigma-Aldrich, Suffolk, UK) treatment (5.0 ng/mL for 48 hours). Control cells were cultured in D-MEM without foetal bovine serum.

Mimics or inhibitors of miR-483-5p or miR-483-3p and the negative control sequence were transfected into human stellate cells or hepatocytes using Lipofectamine 2000 (Invitrogen, Shanghai, China) at a final concentration of 37.5 nmol/L, as previously described. After 6 h, the culture medium was changed. Then, after 48 h, the cells were collected for total RNA and protein extraction.

**Immunofluorescence**

The cells (5×10^5^ cells per chamber) were seeded into 3.5-cm plates. The next day, the cells were rinsed with PBS and fixed with 4% paraformaldehyde for 30 min at room temperature followed by permeabilisation with 0.1% sodium citrate plus 0.1% Triton X-100. The cells were subjected to immunofluorescent staining with an α-SMA, PDGF-β or TIMP2 (1:100) antibody for 16 h at 4°C. The cells were then washed with cold PBS three times for 3 min each and incubated with a secondary antibody (1:400) (Invitrogen) at room temperature for 30 min.

**miRNA quantification**

Total RNA was extracted using TRIzol reagent (Invitrogen, USA). Reverse-transcribed complementary DNA was synthesised with miR-483-5p or miR-483-3p specific stem-loop primers. Subsequently, the cDNA was subjected to real-time PCR on a real-time PCR 7500 system (AB Applied Biosystems, Mannheim, Germany) using SYBR-green PCR Master Mix. U6 were used as internal controls. All PCR primers used were synthesised by the Shengong Company (Shanghai, China). The relative primers see Supplement.

**Quantitative real-time PCR**

Total RNA was extracted using TRIzol reagent (Invitrogen). Reverse transcribed complementary DNA was synthesized with random primers or. Subsequently, the cDNA was subjected to real-time PCR on a 7500 real-time PCR system. GAPDH and were used as internal controls. The primers sequences used were as S2 matrial.

**Western blotting**

Cellular protein extracts were separated in a 8% SDS-polyacrylamide gel and electrophoretically transferred onto a PDVF membrane (Millipore, Bedford, MA, USA). Membranes were blocked overnight with 5% non-fat dried milk and incubated with antibodies. After washing with PBS, the membranes were incubated with horseradish peroxidase-linked secondary antibody. The proteins were visualized using ECL chemiluminescence and exposed to X-ray film. Bands were quantified with Image J (National Institutes of Health, Bethesda, MD, USA).

**miRNA target prediction**

The microRNA target bioinformatics software microRNA.org (http://www.microrna.org/microrna/home.do) was used to predict the miR-483 targets. The details of the complete computational protocol are available at those sites.

**Luciferase activity assay**

The 3’ untranslated region (UTR) of the target mRNAs were amplified by PCR from genomic DNA using cloning primers. The amplicon was cloned into a pMIR-REPORT™ System (ABI, Beijing, China). The third and fourth nucleotides of the miR-22 seed sequence in the 3’ UTR of the targets were mutated using the QuikChange® II Site-Directed Mutagenesis Kit (Agilent Technologies). Each of these constructs was transfected into HEK293T cells together with 50 nM miR-483 mimics, inhibitor or NC, and the pRL-TK vector (Promega, Beijing, China) for the normalisation of the transfection efficiency. The luciferase reporter assay was performed as described.

**Statistical analysis**

The data are presented as the mean ± SE as determined from at least three independent experiments unless otherwise stated. Statistical analyses were performed using the two-tailed Student’s *t* test. *p* < 0.05 was considered significant.

**Supplemental material------Figure**


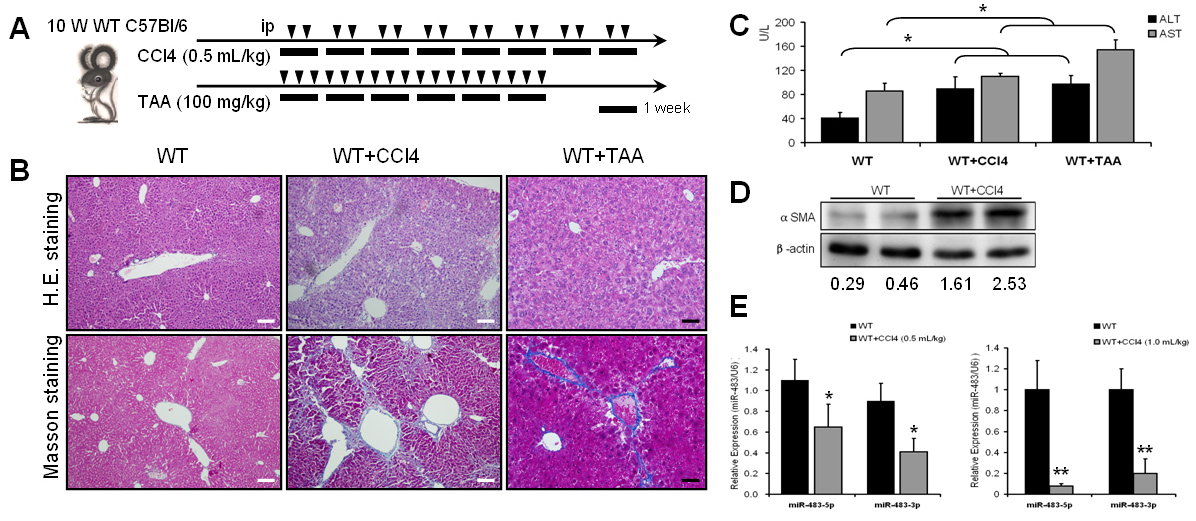


**Fig. S1. Down-regulation of miR-483-5p and miR-483-3p in CCl_4_- or TAA-induced liver fibrosis in mice.** (A) The flowsheet of mouse liver fibrosis induced by CCl_4_ or TAA. Black bar: one week. (B) Liver fibrosis induced by CCl4 (0.5 mL/kg) or TAA was measured by H&E and Masson staining. White bar: 100 μm, Black bar: 50 μm. (C) Serum ALT and AST levels of WT mice induced by CCl_4_ or TAA. (D) Down-regulation of SMA in mouse liver fibrosis. (E) Down-regulation of miR-483 in low and high doses CCl_4_-induced liver fibrosis in mice.


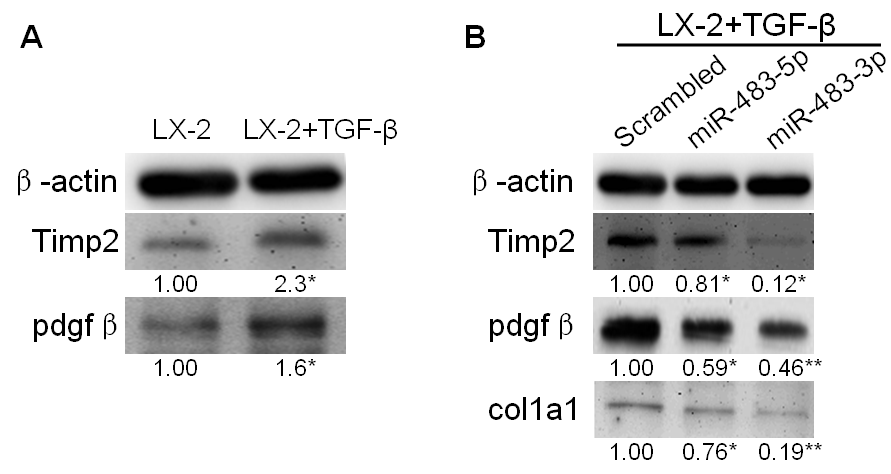


**Fig. S2. The expression of TIMP2 and PDGF at translational level after activition by TGF and overexpress miR-483.** (A) TIMP2 and PDGF were up-regulation after TGF treatment. (B) miR-483 could inhibit the expression of TIMP2 and PDGF in LX-2 cells. (*) p﹤0.05, (**) p﹤0.01.


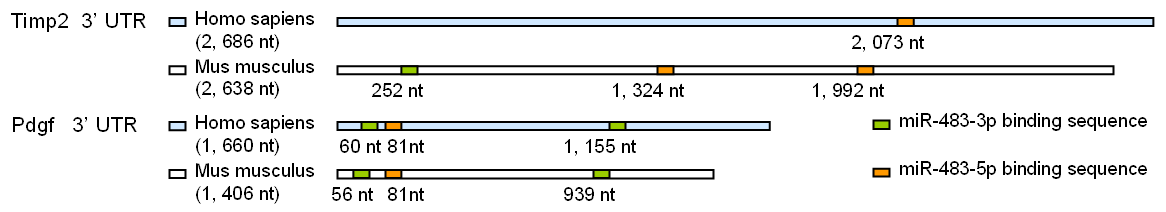


**Fig. S3. The UTRs of TIMP2 and PDGF-β of human and mice.**


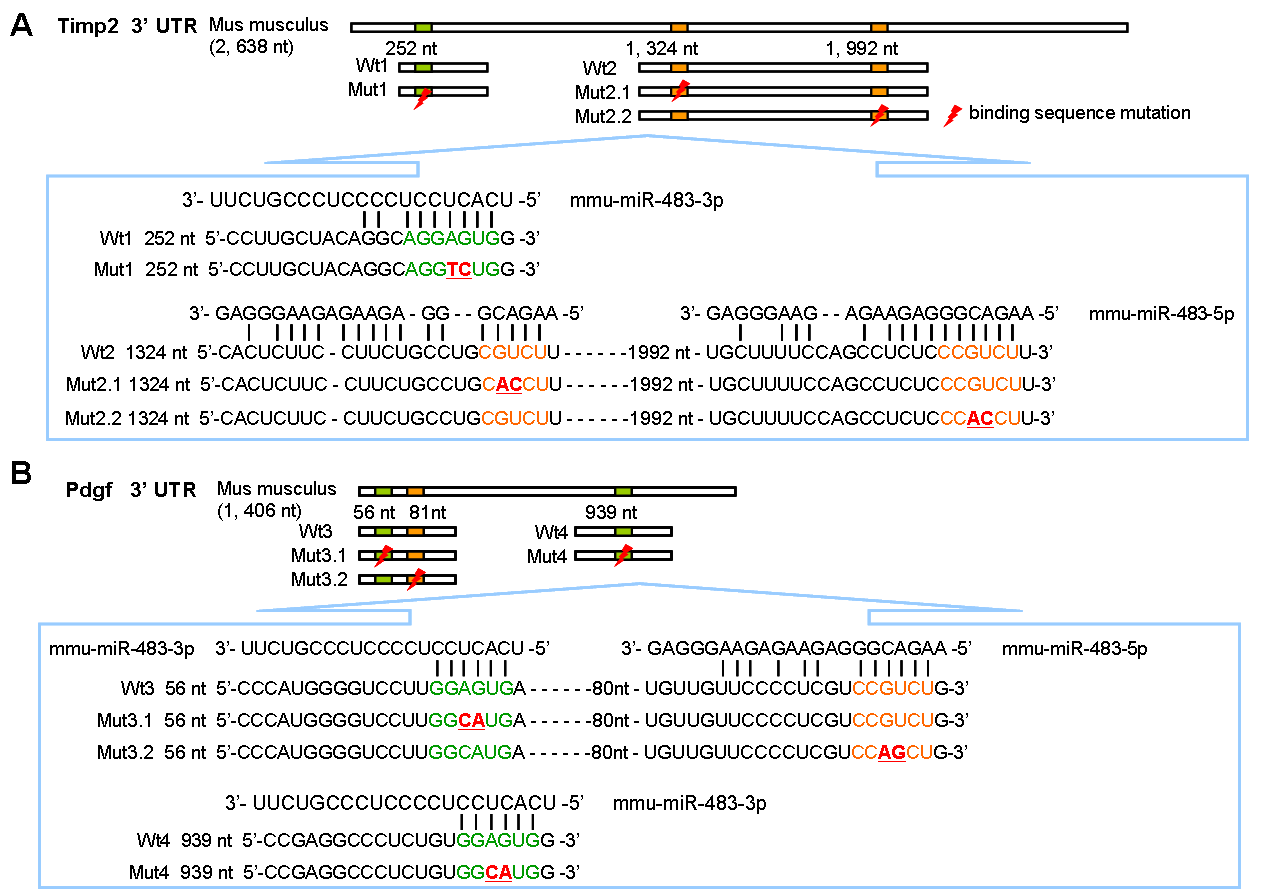


**Fig. S4. Mutant UTR of TIMP2 and PDGF-β.** To investigate the binding of miR-483 and its target, we built the report plasmids containin the wild and mutant UTR of TIMP2 and PDGF.


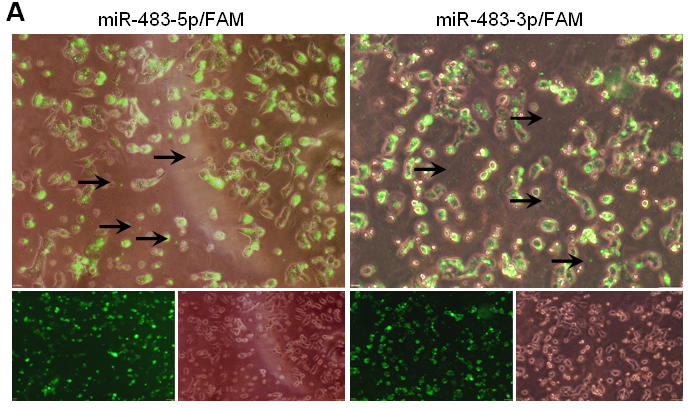


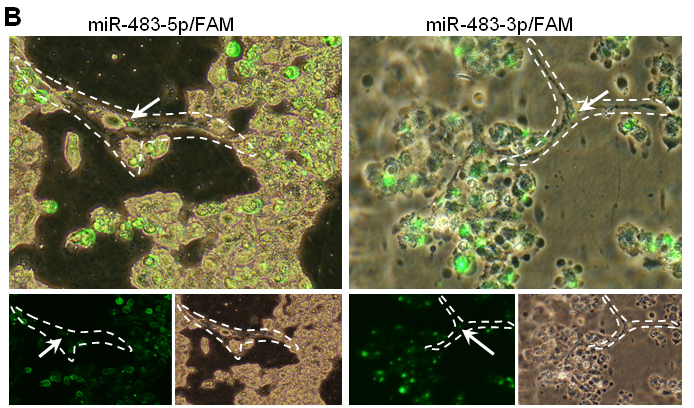


**Fig. S5.** (A) The carboxyfluorescent labelled miR-483 was observed in the cell culture media of HL7702 cells after the culture media was changed 48 h after the transfection and the cells were washed three times with PBS (Black arrowhead: carboxyfluorescent labelled miR-483). (B) The green fluorescence was observed in LX-2 cells, which were direct co-cultured with HL2207 cells transfected with carboxyfluorescent labelled miR-483 (arrowhead: carboxyfluorescent labelled miR-483, dotted line: LX-2 cell).


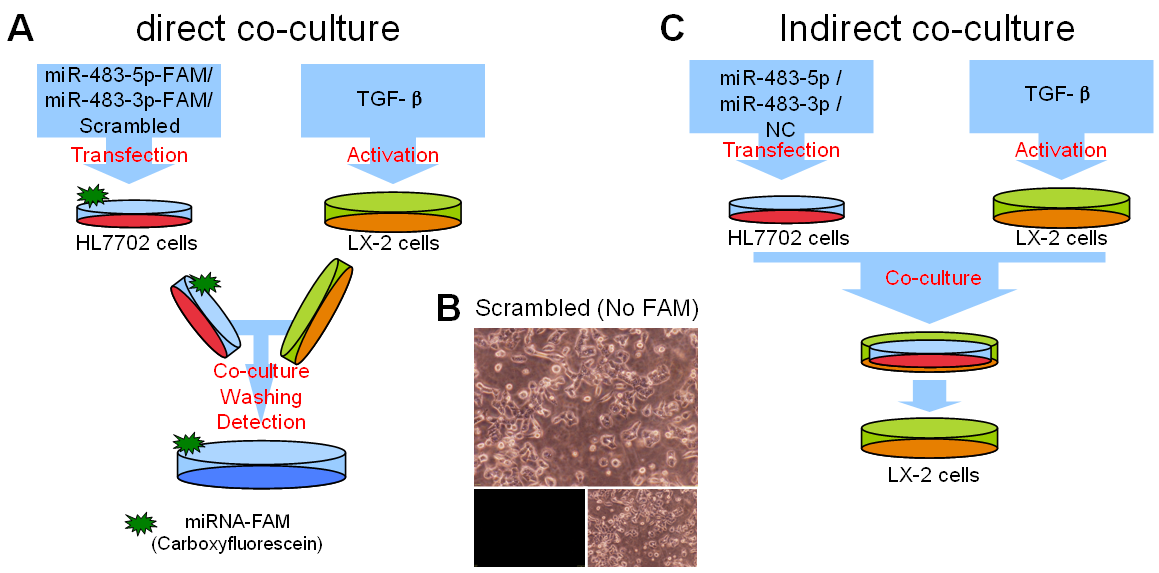


**Fig. S6. Direct and indirect cell co-culture of HL7702 and LX-2.** (A) Direct co-culture of HL7702 with transfection of carboxyfluorescently (FAM) labelled miR-483 and TGF- induced LX-2. The cells were washed with PBS before observation. (B) HL7702 cells were transfected with miR-483 without FAM. (C) Indirect co-culture.


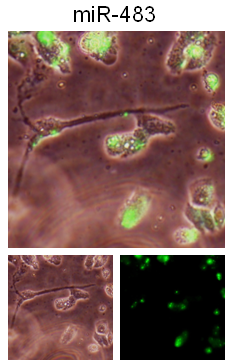


**Fig. S7.** The green fluorescence dot could be observed in LX-2 cells. the lower left: bright field, the lower right: fluorescence.


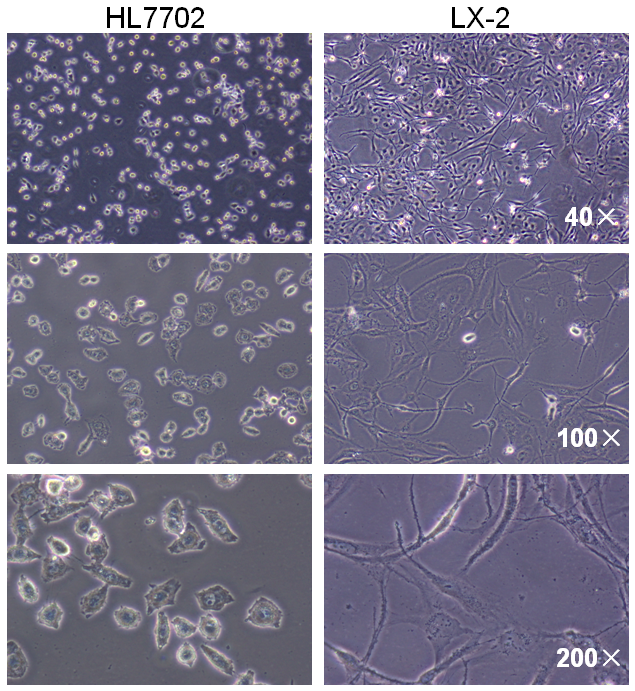


**Fig. S8. The morphology of HL7702 and LX-2 cells.**


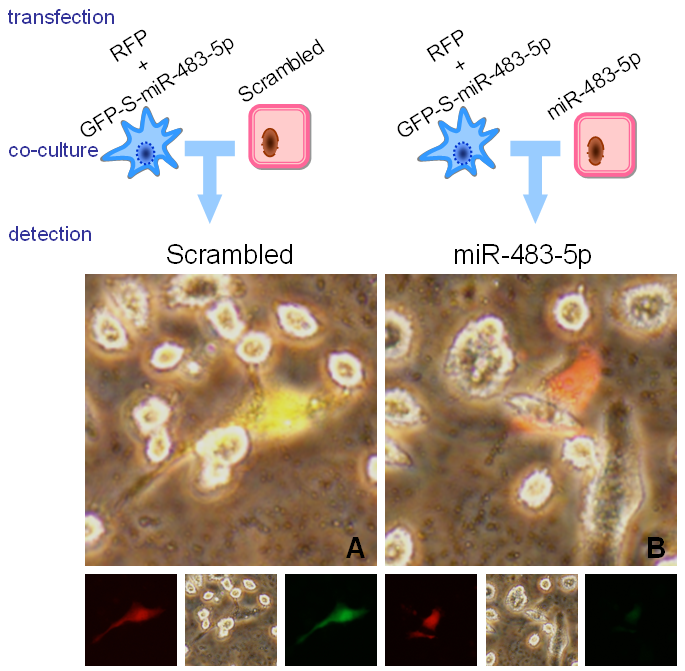


**Fig. S9. Co-culture assay of HL7702 transfected with miR-483-5p (Scrambled) and LX-2 transfected with RFP/GFP-S-483.** The results showed the overexpression of miR miR-483-5p in HL7702 cells reduced the expression of GFP-S-483 plasmids in LX-2 cells, RFP plasmids as internal control. GFP-S-483: GFP-Sponges-miR-483-5p which is a double antisense sequence of miR-483-5p under stream of GFP gene. It serves as an artifial target of miR-483-5p.


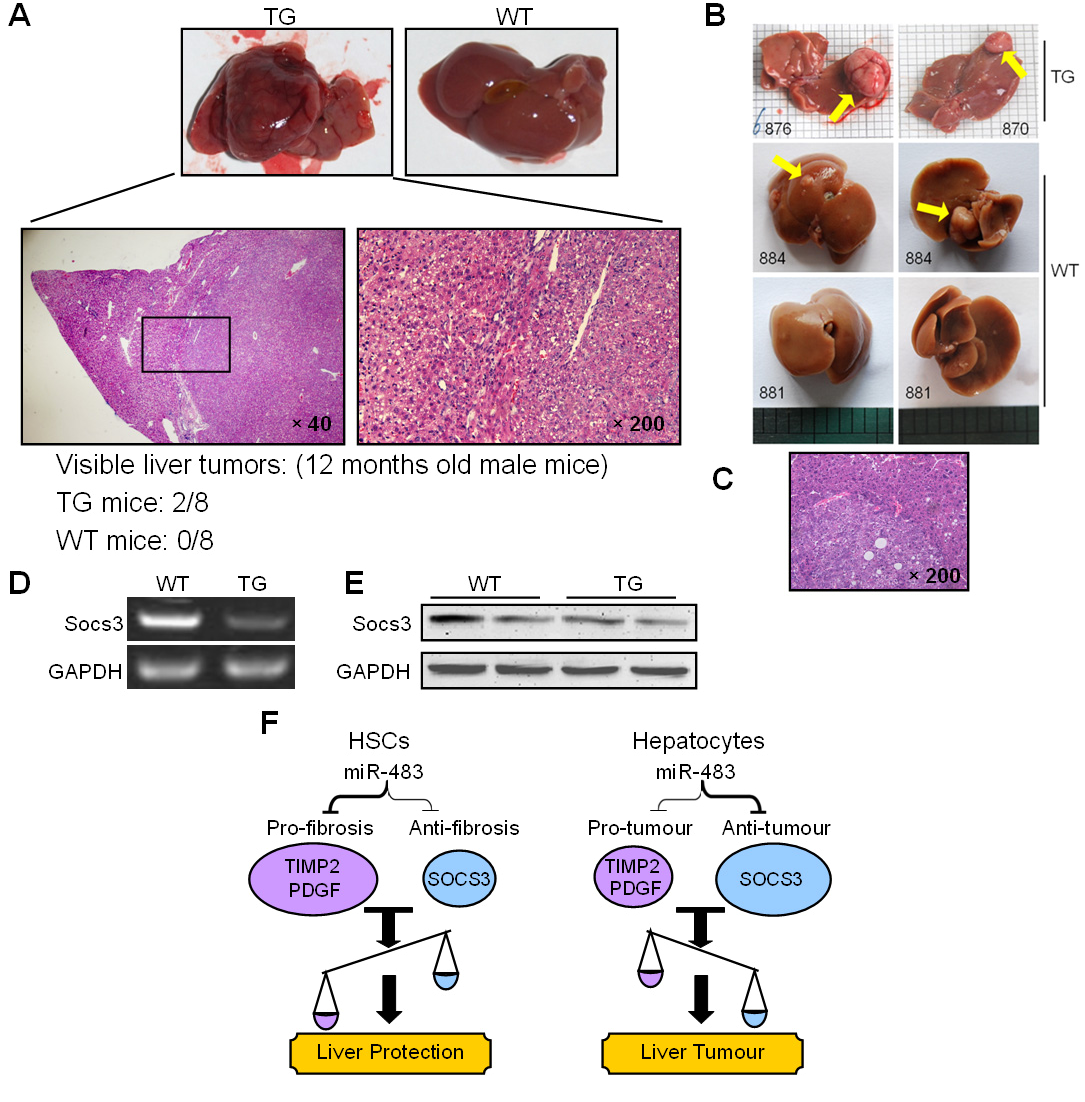


**Fig. S10. Spontaneous tumours in miR-483 transgenic mice.** (A) The overexpression of miR-483 for 12 months promoted the carcinogenesis of HCC. (B) and (C) Overexpression of miR-483 could induce the carcinogenesis induced by DEN. (D) and (E) Socs3 was down-regulated in TG mice liver compared with WT mice.

(F) Therefore, we hypothesise that the dysregulation of miR-483 in liver disease, from liver fibrosis to HCC, depends on the cell type


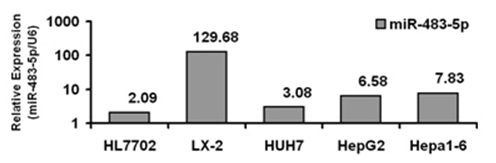


**Fig. S11. Differential expression of miR-483-5p in several cell lines.** Differential expression of miR-483-5p in several cell lines.
