# Supplementary material for: Overexpression of miR-483-5p/3p cooperate to inhibit mouse liver fibrosis by suppressing the TGF-β stimulated HSCs in transgenic mice
Source: J Cell Mol Med. 2014 May 6;18(6):966–74. doi: 10.1111/jcmm.12293 (PMC4508137; doi:10.1111/jcmm.12293)
Supplement: Supplementary file 2 [file jcmm0018-0966-sd2.doc]

**Table. S1. Potential targets of miR-483 predicted by bioinformatics software.**

**(**[**http://www.microrna.org/microrna/home.do**](http://www.microrna.org/microrna/home.do)**)**

**Homo sapiens**

| **Pro-fibrosis** | | |
| --- | --- | --- |
| TIMP2 | TIMP metallopeptidase inhibitor 2 | <http://www.microrna.org/microrna/getMrna.do?gene=7077&utr=18787&organism=9606> |
| PDGFB | platelet-derived growth factor beta polypeptide | <http://www.microrna.org/microrna/getMrna.do?gene=5155&utr=9811&organism=9606> |
| PDGFRB | platelet-derived growth factor receptor, beta polypeptide | <http://www.microrna.org/microrna/getMrna.do?gene=5159&utr=4071&organism=9606> |
| **Anti-fibrosis** | | |
| MMP9 | matrix metallopeptidase 9 | <http://www.microrna.org/microrna/getMrna.do?gene=4318&utr=13379&organism=9606> |

**Mus musculus**

| **Pro-fibrosis** | | |
| --- | --- | --- |
| Timp2 | tissue inhibitor of metalloproteinase 2 | <http://www.microrna.org/microrna/getMrna.do?gene=21858&utr=2244&organism=10090> |
| Pdgfb | platelet derived growth factor, B polypeptide | <http://www.microrna.org/microrna/getMrna.do?gene=18591&utr=6763&organism=10090> |
| Pdgfrb | platelet derived growth factor receptor, beta polypeptide | <http://www.microrna.org/microrna/getMrna.do?gene=18596&utr=5519&organism=10090> |
| Ctgf | connective tissue growth factor | <http://www.microrna.org/microrna/getMrna.do?gene=14219&utr=12694&organism=10090> |
| **Anti-fibrosis** | | |
| Mmp9 | matrix metallopeptidase 9 | <http://www.microrna.org/microrna/getMrna.do?gene=17395&utr=29403&organism=10090> |
