# Supplementary material for: Overexpression of miR-483-5p/3p cooperate to inhibit mouse liver fibrosis by suppressing the TGF-β stimulated HSCs in transgenic mice
Source: J Cell Mol Med. 2014 May 6;18(6):966–74. doi: 10.1111/jcmm.12293 (PMC4508137; doi:10.1111/jcmm.12293)
Supplement: Supplementary file 3 [file jcmm0018-0966-sd3.doc]

**Primers and Sequences**

| **Primers for transgenic mice** | | |
| --- | --- | --- |
| miR483-CL-F | ggtcgaattccact cctgcacgga ggg |  |
| miR483-CL-R | caggtGAATTCcctgt acttggggcccg | 237bp |
| miR483-CO-F | GCCTTCTTCTTTTTCCTACAGCTC |  |
| miR483-CO-R | catcgctgaactgtgacaggaag | 249bp |
| miR483-CO1-F | ctt cctgtcacag ttcagcgatg |  |
| miR483-CO2-R | atgctcaaggggcttcatgatgt | 204bp |
|  |  |  |
| **Primers for quantitation of miRNAs** | | |
| 483-5P Loop-RT-Primer | GTCGTATCCAGTGCGTGTCGTGGAGTCGGCAATTGCACTGGATACGACctccctt |  |
| 483-5p-F | GGAAGACGGGAGAAGAGAAGGG |  |
| 483-5p-R | ATTGCGTGTCGTGGAGTCG |  |
| 483-3P Loop-RT-Primer | GTCGTATCCAGTGCGTGTCGTGGAGTCGGC AATTGCACTGGATACGACaagacgg |  |
| 483-3p-F | AATTTCACTCCTCCCCTCC |  |
| 483-3p-R | ATTGCGTGTCGTGGAGTCG |  |
| U6-RT primer | CGCTTCACGAATTTGCGTGTCAT |  |
| U6-F | GCTTCGGCAGCACATATACTAAAAT |  |
| U6-R | CGCTTCACGAATTTGCGTGTCAT |  |
| **Primers for quantitation of gene expression** | | |
| hsa aSMA F | GACAATGGCTCTGGGCTCTGTAA |  |
| hsa aSMA R | CTGTGCTTCGTCACCCACGTA | 147bp |
| hsa TIMP2 F | GGAGCACTGTGTTTATGCTGGAA |  |
| hsa TIMP2 R | GACCGAGCGATTGCTCAAGA | 135bp |
| mmu aSMA F | TCCCTGGAGAAGAGCTACGAACT |  |
| mmu aSMA R | AAGCGTTCGTTTCCAATGGT | 62bp |
| hsa Col1A1F | CCCGGGTTTCAGAGACAACTTC |  |
| hsa Col1A1R | TCCACATGCTTTATTCCAGCAATC | 148bp |
| hsa PDGFB F | cgctcttcctgtctctctgc |  |
| hsa PDGFB R | Tggtcactcagcatctcataaag | 91bp |
| mmu Pdgfb F | cttccttcctctctgctgctac |  |
| mmu Pdgfb R | Cgctcagcatttcatacagttc | 83bp |
| hsa ACTIN F | TACCTCATGAAGATCCTCACC |  |
| hsa ACTIN R | TTTCGTGGATGCCACAGGAC | 268bp |
| mmu ACTIN F | ccgtaaagacctctatgccaaca |  |
| mmu ACTIN R | Cggactcatcgtactcctgct | 230bp |
| mmu Col1a1 F | AATGGCACGGCTGTGTGCGA |  |
| mmu Col1a1 R | AACGGGTCCCCTTGGGCCTT | 183bp |
| mmu Timp2 F | CCCCCTCTTCAGCAGTG |  |
| mmu Timp2 R | GCGTGTCCCAGGGCACAATGA |  |
| **Primers for lucifersae report plasmids (Wild type and Mutant UTR)** | | |
| **TIMP2** | |  |
| Wt1-F | tggaactagtctgagcactgtgtgtc |  |
| Wt1-R | agagaagcttgcttggaaaggggtgaag | 624bp |
| Mut1-F | GATCCTTGCTACAGGCAGGctTGGAGCACAGACTTGTTAC |  |
| Mut1-R | GTAACAAGTCTGTGCTCCAAGCCTGCCTGTAGCAAGGATC |  |
| Wt2-F | agcaactagtcagccac tcttccttc |  |
| Wt2-R | ggccaagctttgaaccagatacaaaag | 730bp |
| Mut2.1-F | CACTCTTCCTTCTGCCTGCacCTTAAAAAATAAGCTGTCC |  |
| Mut2.1-R | GGACAGCTTATTTTTTAAGgtGCAGGCAGAAGGAAGAGTG |  |
| Mut2.2-F | GCTTTTCCAGCCTCTCCCacCTTTTGTATCTGGTTCAA |  |
| Mut2.2-R | TTGAACCAGATACAAAAGgtGGGAGAGGCTGGAAAAGC |  |
| **PDGFβ** | |  |
| Wt3-F | acga actagt tgctgtattgc ccccatg |  |
| Wt3-R | agag aagctt tgtggccac ctgcccaga | 520bp |
| Mut3.1-F | GCCCCCATGGGGTCCTTGGcaTGATAATGTTGTTCCCCTC |  |
| Mut3.1-R | GAGGGGAACAACATTATCAtgCCAAGGACCCCATGGGGGC |  |
| Mut3.2-F | GTTGTTCCCCTCGTCCagCTGTCTCGATGCCTG |  |
| Mut3.2-R | CAGGCATCGAGACAGctGGACGAGGGGAACAAC |  |
| Wt4-F | tcaa actagtgagg aaagggaacc ttgttt |  |
| Wt4-R | tccc aagcttccaatctactggctagcttc |  |
| Mut4-F | GAGGCCCTCTGTGGcaTGGCTGTGTGTGCC |  |
| Mut4-R | GGCACACACAGCCAtgCCACAGAGGGCCTC |  |
| **PDGFβR** | |  |
| Wt5-F | gtct actagtgc aactgcccagacctt |  |
| Wt5-R | ctcc aagctt ggagatgccagctagcca | 444bp |
| Mut5-F | GCCCAGACCTTGACTTGGcaTGACAGTGAGTGTCCTAG |  |
| Mut5-R | CTAGGACACTCACTGTCAtgCCAAGTCAAGGTCTGGGC |  |
|  |  |  |

Has-miR-483-5p mimics

FAM-AAGACGGGAGGAAAGAAGGGAG

Has-miR-483-3p mimics

FAM-UCACUCCUCUCCUCCCGUCUU

FAM: carboxyfluorescein

AUGC: 2´-OME
